# Ecology of West Nile Virus in the Danube Delta, Romania: Phylogeography, Xenosurveillance and Mosquito Host-Feeding Patterns

**DOI:** 10.3390/v11121159

**Published:** 2019-12-14

**Authors:** Alexandru Tomazatos, Stephanie Jansen, Stefan Pfister, Edina Török, Iulia Maranda, Cintia Horváth, Lujza Keresztes, Marina Spînu, Egbert Tannich, Hanna Jöst, Jonas Schmidt-Chanasit, Daniel Cadar, Renke Lühken

**Affiliations:** 1 Bernhard Nocht Institute for Tropical Medicine, WHO Collaborating Centre for Arbovirus and Hemorrhagic Fever Reference and Research, 20359 Hamburg, Germany; alex_tomazatos@yahoo.com (A.T.); jansen@bnitm.de (S.J.); maranda.iulia@gmail.com (I.M.); tannich@bnitm.de (E.T.); joest@bnitm.de (H.J.);; 2 Humboldt-Universität zu Berlin, 10117 Berlin, Germany; stefan.pfister@hu-berlin.de; 3 “Lendület” Landscape and Conservation Ecology, Institute of Ecology and Botany, MTA Centre for Ecological Research, 2163 Vácrátót, Hungary; torok.edina@okologia.mta.hu; 4 Department of Clinical Sciences-Infectious Diseases, University of Agricultural Sciences and Veterinary Medicine, 400372 Cluj-Napoca, Romaniamarina.spinu@gmail.com (M.S.); 5 Center of Systems Biology, Biodiversity and Bioresources, Faculty of Biology and Geology, Babeș-Bolyai University, 400372 Cluj Napoca, Romania; keresztes2012@gmail.com; 6 German Centre for Infection Research (DZIF), partner site Hamburg-Luebeck-Borstel-Riems, 20359 Hamburg, Germany; 7 Faculty of Mathematics, Informatics and Natural Sciences, Universität Hamburg, 20148 Hamburg, Germany

**Keywords:** West Nile virus, virus genetics, phylogeography, xenosurveillance, blood meal

## Abstract

The ecology of West Nile virus (WNV) in the Danube Delta Biosphere Reserve (Romania) was investigated by combining studies on the virus genetics, phylogeography, xenosurveillance and host-feeding patterns of mosquitoes. Between 2014 and 2016, 655,667 unfed and 3842 engorged mosquito females were collected from four sampling sites. Blood-fed mosquitoes were negative for WNV-RNA, but two pools of unfed *Culex pipiens* s.l./*torrentium* collected in 2014 were tested positive. Our results suggest that Romania experienced at least two separate WNV lineage 2 introductions: from Africa into Danube Delta and from Greece into south-eastern Romania in the 1990s and early 2000s, respectively. The genetic diversity of WNV in Romania is primarily shaped by in situ evolution. WNV-specific antibodies were detected for 19 blood-meals from dogs and horses, but not from birds or humans. The hosts of mosquitoes were dominated by non-human mammals (19 species), followed by human and birds (23 species). Thereby, the catholic host-feeding pattern of *Culex pipiens* s.l./*torrentium* with a relatively high proportion of birds indicates the species’ importance as a potential bridge vector. The low virus prevalence in combination with WNV-specific antibodies indicate continuous, but low activity of WNV in the Danube Delta during the study period.

## 1. Introduction

Emerging or re-emerging mosquito-borne viruses (moboviruses) are of growing concern in Europe [1]. Several moboviruses circulate on the European continent [2]. Thereby, West Nile virus (WNV, genus *Flavivirus,* family *Flaviviridae*) is of particular importance. This zoonotic virus belongs to the Japanese encephalitis serocomplex and is one the most widespread moboviruses in the world [3,4,5]. Enzootic transmission takes place between birds as amplifying hosts and mosquitoes as vectors. WNV can cause high mortalities in birds, but spillover events also have significant public health consequences, e.g., headache, rash and even neurological complication [6,7]. Various outbreaks of WNV infections have been reported in southern and southeastern Europe, resulting in several thousand human cases with dozens of fatal outcomes [8,9]. Thereby, Romania is a hotspot for WNV circulation [6,8,10,11,12,13,14]. Over the last two decades, the country has experienced at least three large outbreaks of WNV (1996, 2010, 2018) with a mortality rate of up to 20%. Genetic and phylogenetic analyses grouped the WNV strains into eight distinct evolutionary lineages, from which the most spread worldwide and associated with disease and outbreaks belong to lineages 1 and 2 [15,16]. The virus is transmitted and maintained in the natural cycle by mosquitoes (mostly of the *Culex* genus) as vectors with birds as the main amplifying hosts, while humans and horses are considered incidental or dead-end hosts. Nowadays, West Nile virus exhibits a worldwide distribution throughout Africa, the Middle East, Europe, western Russia, southwestern Asia, and Australia [15]. Starting early 1990s, the frequency, severity and geographic range of human WNV outbreaks increased with the appearance of new viral strains in Romania, Russia, Israel, and Greece [17,18]. In the western hemisphere, West Nile virus spread from its 1999 appearance in New York City throughout the Pacific Coast and Argentina in 2005 [19,20,21]. Nowadays, the severity, magnitude and geographic location of the WNV outbreaks differs greatly, being instrumented by the local ecological conditions and increased anomalies of seasonal temperature. The Danube Delta Biosphere Reserve (DDBR) is the second largest wetland in Europe. This complex of ecosystems is predominantly located in Romania, with small parts also located in the Ukraine. The heterogeneous area of the DDBR has a high biodiversity with an important function as a major hub for bird migration [22,23]. Thus, the area has a high risk for the introduction of zoonotic pathogens. Introduced moboviruses find an abundant and diverse mosquito fauna [9,23]. Most of the DDBR is only accessible by boat. This makes comprehensive studies on mosquito fauna and associated viruses a difficult task, e.g., showcased by a recent pilot study, detecting two new mosquito species for Romania (*Aedes hungaricus* and *Anopheles algeriensis*) [9].

The circulation of WNV in the DDBR was reported before 2014 [12,24]. WNV dynamics in the Danube Delta are positively correlated with temperature and negatively correlated with rainfall. However, there is still a lack of knowledge driving the risk of WNV transmission under near-natural conditions as present in the DDBR. Therefore, in this study, classical virus screening of mosquitoes was combined with a xenosurveillance approach. Testing for WNV-specific antibodies in sentinel horses or chicken is a common monitoring tool in Europe [25]. However, such a surveillance system is difficult to implement under the remote conditions of a wetland system and the results might not reflect the natural transmission cycle. In addition, comprehensive sampling from wild animals needs a lot of effort. In this study, mosquitoes were used as “biological syringes”, i.e., blood-fed specimens were screened for WNV-specific antibodies and viral RNA. Experimental studies by Leigthon et al. [26] demonstrated the potential of mosquitoes for sero-epidemiological studies. This was further supported by a field-study in Thailand detecting antibodies against dengue virus and Japanese encephalitis virus in blood-fed mosquitoes in two different mosquito species [27]. However, a broad application of this method for different wild mosquito species, which feed on different vertebrate hosts, was missing.

Host-feeding patterns of blood-sucking arthropods shape the transmission cycle of vector-borne pathogens, offering direct insights into the interaction between vectors and hosts. However, there is still a lack of knowledge about the host spectrum of mosquitoes in Europe [28]. Previous studies predominantly investigated certain combinations of vector species and pathogens, e.g., *Culex* spp./WNV [29,30,31,32], *Culex* spp./avian malaria [33], *Anopheles* spp./malaria [33,34] and *Aedes albopictus* as an invasive vector species for a variety of pathogens [35]. Only few European studies analyzed the host-feeding patterns of a wide range of species [36,37,38], which is required to better understand pathogen circulation.

Thus, in order to get comprehensive insight into the ecology of WNV in the DDBR, the mosquito fauna was studied in a longitudinal surveillance program over three years. Molecular assays were applied to (i) screen for WNV infections in mosquitoes, analyze the evolutionary mechanism of the virus and its dispersal patterns in Europe, in particular in Romania and the DDBR, (ii) detect WNV-specific antibodies in the blood meals from horses, dogs, humans and birds and (iii) identify potential vector species by analyzing the host-feeding patterns of the blood-fed mosquitoes.

## 2. Materials and Methods

Mosquitoes were collected within a longitudinal arbovirus surveillance program between 2014 and 2016 at two sampling sites in a rural/urban environment (Letea, Sulina) and two near-natural sampling sites (Dunărea Veche and Lake Roșuleț) in the DDBR. Each year, on average, every tenth day between April and September, three to four (2014, 2015) or one (2016) carbon dioxide-baited Heavy Duty Encephalitis Vector Survey trap(s) (Bioquip Products Inc., CA, USA) were installed at each site. A detailed description of the collection sites can be found in Török et al. [9]. The DDBR Authority issued research permits (9/25.04.2014, 10692/ARBDD/25.04.2014; 7717/ARBDD/28.04.2016, 11/28.04.2016). The collected specimens were transported on dry ice, stored in the freezer and identified by morphology on chill tables using a stereomicroscope (Olympus SZX12, Tokyo, Japan) [39]. Blood-fed mosquitoes were separated from unfed specimens. Furthermore, morphologically identified *Culex pipiens* specimens were typed to species level (*Cx. pipiens pipiens* f. *pipiens*, *Cx. pipiens pipiens* f. *molestus* or *Cx. torrentium*) using a molecular assay [40].

For the WNV screening, mosquito pools between 1 and 250 specimens were pooled per sampling site and sampling date. Mosquitoes were put in 2 mL safe-lock tubes (Eppendorf, Hamburg, Germany) or 50 mL centrifuge tubes (Sarstedt, Nümbrecht, Germany) with zirconia beads (2 mm, BioSpec Products, Bartlesville, OK, USA) and 0.5 or 3 mL chilled high-glucose (4.5g/L) Dulbecco’s modified Eagle’s medium (DMEM) (Sigma-Aldrich, St. Louis, MO, USA). Mosquitoes were homogenized in a TissueLyser or TissueLyser II (Qiagen, Hilden, Germany) for 2 min at 30–50 Hz. The suspension was clarified by centrifugation for 1 min at 8000 rpm and 4 °C. RNA was extracted with a KingFisher Flex 96 Deep-Well Magnetic Particle Processor using the MagMAX CORE Nucleic Acid Purification Kit (ThermoFisher Scientific, Waltham, MA, USA). Samples were tested with pan-flavivirus RT-PCR modified from Chao et al. [41] as described in detail by Becker et al. [42]. WNV-positive mosquito pools were subjected to Sanger sequencing (LGC Genomics, Berlin, Germany) for complete genome sequencing [43].

The blood-fed specimens were individually placed into 2 mL safe-lock tubes. Homogenization and extraction were conducted using the same protocol as described above. Thereby, 30 μL supernatant from each of ten specimens was pooled for WNV screening. Detection of WNV-RNA was conducted with the RealStar WNV RT-PCR Kit 1.0 (altona Diagnostics, Hamburg, Germany).

For the host identification, the supernatant of individual blood-fed specimens was heat-inactivated at 99 °C for 1 min in a Peqlab thermocycler (VWR International GmbH, Darmstadt, Germany) for the reduction of possible inhibitors. The PCR assay used the Phusion Blood Direct Master Mix (Thermo Fisher Scientific, MA, USA), 5 μL of the homogenate was used in a total of 30 μL reaction volume for PCR amplification of the cytochrome b gene [44,45]. Amplification was conducted by incubation for 5 min at 98 °C, followed by 40 cycles of 1 s at 98 °C, 5 s at 57 °C and 30 s at 72 °C, ending with incubation for 1 min at 72 °C. If the reaction with the first primer set yielded no result, the PCR reaction was repeated using another pair of vertebrate-specific primers targeting the 16S rRNA gene fragment [46]. The same applied to potential mixed blood meals, as indicated by double peaks in the sequence electropherograms at different positions, resulting in unreadable chromatograms. For this PCR, amplification was conducted by incubation for 5 min at 98 °C, followed by 40 cycles of 1 s at 98 °C, 5 s at 50 °C and 30 s at 72 °C, concluded by incubation for 1 min at 72 °C. The amplicons were sequenced (LGC Genomics, Berlin, Germany) and analyzed with Geneious v9.1.7 (Biomatters, Auckland, New Zealand). Sequences were compared to available sequences from GenBank database (https://blast.ncbi.nlm.nih.gov/). Host species were identified if the percentage identity was 95% or higher. The statistical computer program R [47] was used for all data analyses. Data manipulation and visualization was conducted with functions from the packages plyr [48], dplyr [49], magrrittr [50] and ggplot2 [51]. Spearman’s rank correlation was used to analyze the statistical relationship between the number of analyzed specimens per mosquito species and the number of detected host species. For each mosquito species, higher order taxa (e.g., Anatidae, Bovidae, Chiroptera) were only considered for the calculations of host species, if no corresponding taxa of lower ranks were detected. The frequencies of detected birds, non-human mammals or humans between the six most abundant mosquito species and between the four sampling sites were compared with Chi-square tests with Bonferroni corrected p-values for multiple pairwise comparisons.

Horse-, human-, dog- and bird-derived blood meals were tested for WNV-specific IgG/IgY, using an indirect immunofluorescence (IIF) assay as described previously [52]. Host species were selected, which are important amplifying hosts (bird), known to become critically ill from WNV infections (human, horse) or were previously identified to be suitable sentinel species for WNV (dog, horse) [6,7,53,54]. In brief, Vero cells infected with WNV NY99 were seeded on microscope slides with 12 reaction wells (Marienfeld, Lauda-Königshofen, Germany). Slides were treated with Acetone (99%), 15 µL of each sample (single mosquito homogenized in 500 µL) was transferred into one reaction well. Cells were washed with PBS and stained with Alexa Fluor^®^ 488-conjugated Alpaca Secondary Antibodies (Jackson ImmunoResearch, West Grove PA, USA 1:200 in 1% Evans blue solution), namely goat anti-human IgG, goat anti-horse IgG, rabbit anti-chicken IgY and rabbit anti-dog IgG antibodies, depending on the identified blood-meal source. In order to test for cross-reactivity with heterologous flaviviruses potentially circulating in the sampling area, the WNV IgG positive samples were also tested for Usutu virus- (USUV) and tick-borne encephalitis virus- (TBEV) specific IgG using the same assay with the respective virus.

Genomes obtained for WNV strains from Danube Delta were compared with all complete and partial publicly available NS5 gene sequences from Europe and Africa. Phylogenetic trees were inferred using the Bayesian Markov chain Monte Carlo (MCMC) approach available in BEAST v1.10 [55]. Analyses were performed under the best fit nucleotide substitution model identified as the GTR +Γ for complete genome and TN93+Γ for partial NS5 datasets using jModelTest 2 [56] and a prior MCMC was chosen by testing all models and determining Bayes factors (log_10_ BF). We employed TempEst for an interactive regression approach to explore the association between genetic divergence through time and sampling dates [57]. In order to assess the spatial temporal dynamics of WNV, the time to most recent common ancestor (tMRCA), and the effective population dynamics of WNV, we employed a relaxed uncorrelated log normal (UCLN) molecular clock, a flexible demographic model (coalescent Gaussian Markov Random field Bayesian Skyride model, GMRF) as the best demographic scenario detected. In all cases, each of the MCMC chain lengths was run for 10^8^ generations (with 10% burn-in) and subsampled every 10^4^ iterations to achieve convergence. The Bayesian maximum clade credibility (MCC) trees were visualized using FigTree v1.4.1 (http://tree.bio.ed.ac.uk/software/figtree/). To test the hypothesis that WNV is periodically imported from Africa into Europe, a phylogeographic analysis was conducted using a discrete model attributing state characters represented by the detection locality of each strain and the Bayesian stochastic search variable (BSSV) algorithm implemented in BEAST v1.10 [55].

## 3. Results

### 3.1. Mosquitoes and WNV in the Danube Delta

In total, 655,667 mosquitoes representing 14 species and four unspecified taxa (unidentified, *Aedes* spp., *Culex* spp. and *Anopheles* spp.) were collected (Table S1). The mosquitoes were dominated by six species: *Coquilettidia richiardii* (57.9%), *Anopheles hyrcanus* (24.8%), *Anopheles maculipennis* s.l. (4.3%), *Aedes caspius* (3.9%), *Culex pipiens* s.l./*torrentium* (3.5%) and *Aedes vexans* (2.5%). Other mosquito species were represented by 0.0002% to 2.3% individuals per taxa. WNV-RNA was detected in two pools of unfed *Cx. pipiens* s.l./*torrentium* specimens, while all blood-fed mosquito specimens were tested negative. Both WNV-positive pools were collected in the second half of June 2014 at Lake Roșuleț (4 specimens), a near-natural site, and Sulina (95 specimens), the only town in the DDBR.

### 3.2. Genome Characterization of WNV in the DDBR

Both WNV positive mosquito pools have been subjected to Sanger sequencing for complete genome sequencing as described elsewhere [43] and deposited in GenBank under the accession numbers MH939153 and MH939154. Sequence comparison between the two sequenced genomes revealed 51 nucleotide (identity rate 99.5%) and 8 amino acid (identity rate 99.8%) differences almost all of them distributed along the polyprotein (Figure S1). Several structural and nonstructural genes of the WNV from Danube Delta exhibited unique or similar amino acid changes exclusively with African WNV strains (Figure S2).

### 3.3. Phylogeography and Spatio-Temporal Dispersal Pattern of WNV

**Figure 1:**
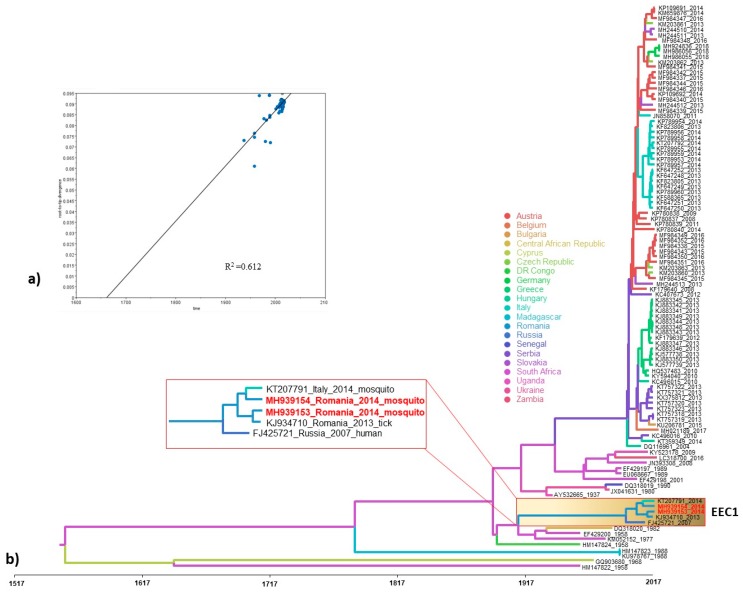
(**a**) Root-to-tip regression analysis of the West Nile virus (WNV) complete genome based maximum likelihood tree. Plots of the root-to-tip genetic distance against sampling time are shown; (**b**) Bayesian maximum clade credibility (MCC) tree representing the time scale phylogeny of WNV lineage 2 based on complete genome sequences, including the EEC1 clade. The colored branches of the MCC tree represent the most probable geographic location of their descendant nodes (see color codes). Time is reported in the axis below the tree and represents the year before the last sampling time (2018).

**Figure 2:**
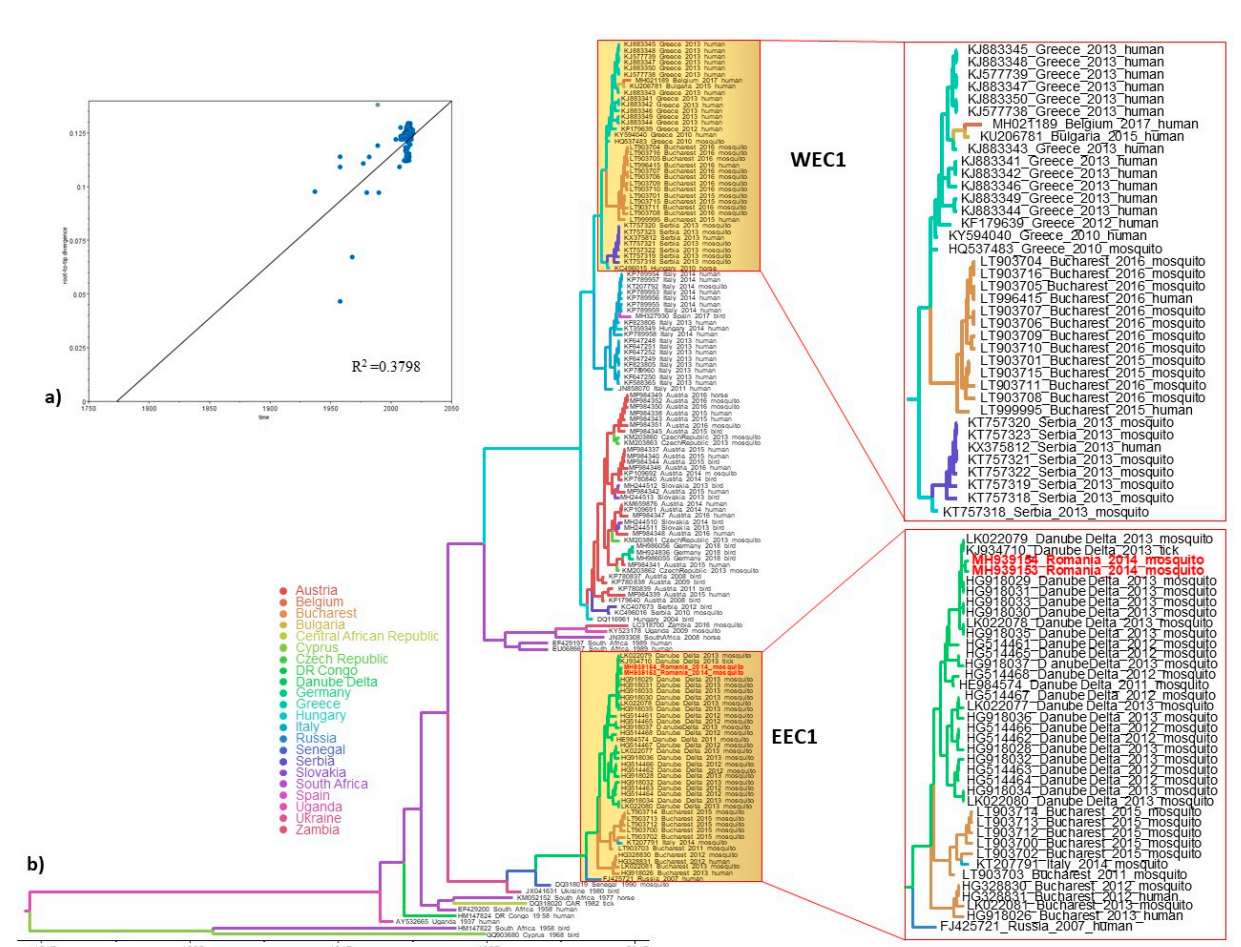
(**a**) Root-to-tip regression analysis of the WNV partial NS5 based maximum likelihood tree. Plots of the root-to-tip genetic distance against sampling time are shown; (**b**) Bayesian maximum clade credibility (MCC) tree representing the time scale phylogeny of WNV lineage 2 based on NS5 gene sequences, including the EEC1 and WEC1 clades. The colored branches of the MCC tree represent the most probable geographic location of their descendant nodes (see color codes). Time is reported in the axis below the tree and represents the year before the last sampling time (2018).

**Figure 3:**
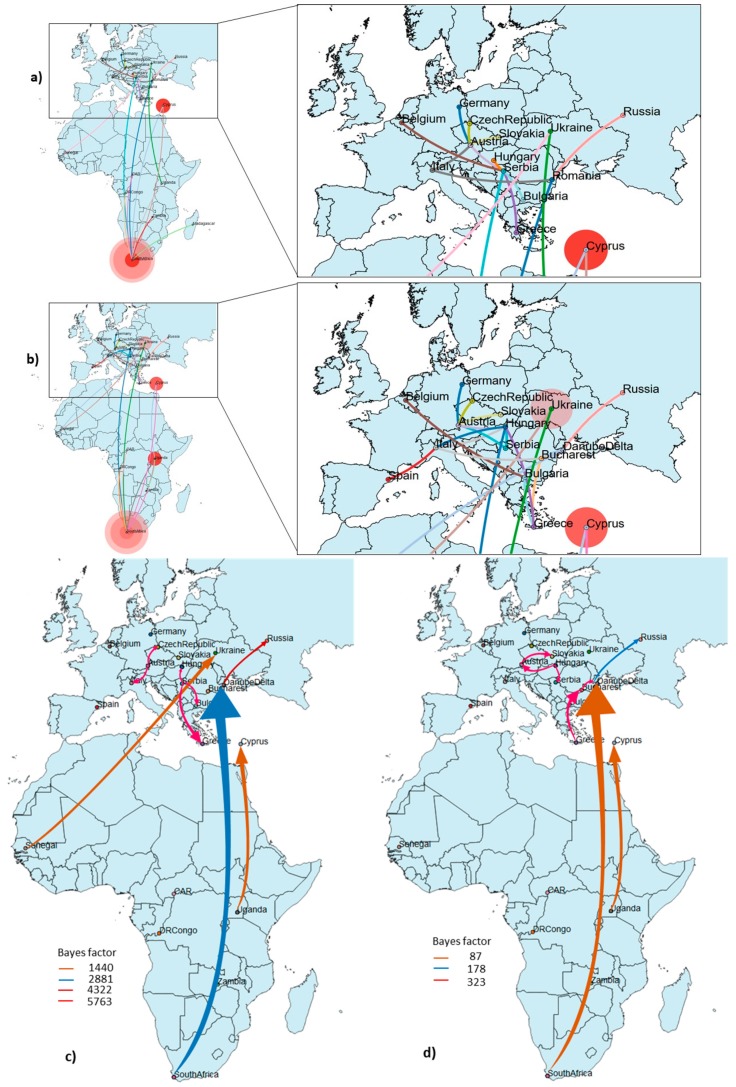
Spatial dynamics of the WNV lineage 2 reconstructed from the (**a**) complete genome and (**b**) partial NS5 based on MCC tree, a flexible demographic prior (coalescent Gaussian Markov Random field Bayesian Skyride model, GMRF) with location states and a Bayesian Stochastic Search Variable Selection (BSSVS) with location states. The directed lines between locations connect the sources and target countries (color coded) of viral strains and represent branches in the MCC tree along which the relevant location transition occurs. Location circle diameters are proportional to the square root of the number of MCC branches maintaining a particular location state at each time-point. Migration pattern of WNV between Africa and Europe and within Europe based on Bayes factor (BF) test for significant non-zero rates using complete genome (**c**) and partial NS5 dataset (**d**). Viral migration patterns are indicated between the different regions and countries and are proportional to the strength of the transmission rate (Bayes factor [BF]). The color of the connections indicates the origin and the direction of migration and are proportional with the strength of connections. Only well supported paths between locations are shown.

The Bayesian phylogenetic tree of the complete coding sequence of WNV showed that the strains from Danube Delta clustered in the Eastern European clade 1 together with the strains Hyalomma/Romania/2013, Volgograd/2007 and Italy 792/14 (Figure 1). Given that the majority of available sequences from Romania are partial NS5 gene fragments, we have inferred Bayesian MCC phylogenies with similar topologies as for the complete genome-based tree. In addition, the phylogenies revealed that the Romanian WNV strains fell into two distinct monophyletic clades within WNV phylogeny, suggesting two distinct introductions into Romania (Figure 2b). One clade designated as Eastern European clade 1 (EEC1) included all WNV strains from Danube Delta and some from south-east Romania (Bucharest), while the second clade designated as Western European clade 1 (WEC1), which forms also a distinct monophyletic clade with WNV strains from south-east Romania, but not from DDBR (Figure 1b, Figure 2b). To assess the viral migration and origin of the WNV in Romania, a discrete-trait phylogeography analysis [58] using the complete genome and NS5 datasets was used to reconstruct the WNV movements between continents/countries. Both datasets exhibited a strong temporal signal and the coefficient of rate variation supported the use of a relaxed clock model (Figure 1a, Figure 2a). The phylogenetic analysis revealed that the long-distance movement pattern of WNV between Africa and Europe occurred. We estimated at least 6 intercontinental and 10 continental (Europe) viral migration events (Figure 3). For Romania, we observed at least 2 distinct introduction events (Figure 1b, Figure 2b). The limited number of available sequences from Romania and the lack of WNV data from several European and African countries make it difficult to infer with confidence the spatiotemporal pattern of WNV EEC1. The time to the most recent common ancestor (tMRCA) of the Romanian WNV strains from WEC1 and EEC1 clades indicates a very recent emergence. These strains were most likely introduced into Romania during the 1990s and 2000s as two distinct introduction events (Figure 1, Figure 2 and Figure 3). The EEC1 clade seems to be a descendant of an ancestor that probably emerged in South Africa around 1910 (95% HPD for 1901–1920; posterior probability 0.96) (Figure 1b), while the WEC1 clade shares a common ancestor that probably emerged in Greece around 1999 (95% HPD for 1994–2003; posterior probability 0.99) (Figure 2b). The spatial origin and diffusion patterns of the WNV were reconstructed using a BSSV analysis. The earliest introduction and migration event of WNV lineage 2 in Romania (Danube Delta) was detected from South Africa (analysis based on complete genome) or Senegal (based on NS5) between 1992 and 2001, after which the virus dispersed to Russia, Italy and southeast Romania (Bucharest) (Figure 1, Figure 2 and Figure 3). The second origin and introduction of WNV in Romania was detected to be from Greece (based on NS5) between 2001 and 2002 (Figure 1, Figure 2 and Figure 3). Furthermore, the phylogeographic analysis also revealed the co-circulation of both EEC1 and WEC1 in south-east Romania, but not in Danube Delta (Figure 1, Figure 2 and Figure 3).

The analysis of the complete genome sequences revealed nonsynonymous geographic and clade-specific mutations in all members of EEC1 including some African ancestral specific amino acid residues which further strengthen the African origin of the WNV circulating in Danube Delta (Figure S2).

### 3.4. Screening for WNV-Specific IgG Antibodies

Nineteen blood meals (2.2%, n = 858 analyzed mosquito specimens) contained WNV-specific antibodies (Table 1, Table S2). Seven of these samples originated from dogs (6.3%, n = 111) and 12 from horses (3.1%, n = 391). All blood meals from birds (n = 85) and humans (n = 271) were WNV IgY/IgG negative. Positive samples were detected for all four sampling sites. WNV IgG positive samples were also tested for USUV- and TBEV-specific IgG. Only one WNV IgG positive blood meal from a dog was also tested positive for USUV-specific IgG.

### 3.5. Host-Feeding Patterns

From the total mosquito collection, 3842 mosquitoes (0.6%) were blood-fed, belonging to 13 mosquito species (Table 2, Table S3). The blood-fed mosquito species were dominated by six species: *An. hyrcanus* (37.8%), *Cq. richiardii* (27.4%), *An. maculipennis* s.l. (14.8%), *Ae. vexans* (8.9%), *Ae. caspius* (6.1%) and *Cx. pipiens* s.l./*torrentium* (2.3%). Other mosquito species were represented by 1 (0.03%) to 36 (0.94%) specimens per taxon (Table S3). Fifty-one of the collected 88 blood-fed specimens of *Cx. pipiens* s.l./*torrentium* were identified as *Cx. pipiens pipiens* f. *pipiens*.

The success rate for the identification of the blood sources was 60.7% (2331 specimens), amounting to 2348 identified hosts from 43 species and three unspecified taxa (Anatidae, Bovidae, Chiroptera). The difference of 17 specimens between the number of detected hosts and analyzed mosquito specimens results from mixed blood-meals (Table 2). Hosts were detected for 12 (92.3%) out of the 13 analyzed blood-fed mosquito species, with no successful PCR amplification for a single specimen of *Culex martinii*. The largest number of host taxa was detected for *Cq. richiardii* (30 species, 827 successfully analysed specimens), followed by *Cx. pipiens* s.l./*torrentium* (17 species, 56 specimens), *An. maculipennis* s.l. (15 species, 280 specimens) and *An. hyrcanus* (13 species, 791 specimens). Both, *Ae. vexans* and *Ae. caspius* fed on a moderate number of host species (nine host species each, 230 and 106 specimens) (Table S3). The other mosquito species with two to 18 specimens fed on two to five host species. Not surprisingly, the number of collected specimens and detected host species were statistically significantly correlated (Spearman rho = 0.90, *p* < 0.001).

Cattle (*Bos taurus*) were the most common host (n = 1009, 43.0% of the detected hosts), followed by horse (*Equus caballus*, n = 391, 16.7%), wild boar (*Sus scrofa*, n = 382, 16.3%), humans (n = 271, 11.5%) and dog (n = 111, 4.7%) (Table 2). The non-human mammalian host group (19 host species and two taxa of higher order) was the most numerous group (n = 1992, 85.0% of the detected hosts), followed by humans (n = 271, 11.5% of all collected mosquitoes). Birds represented the smallest (n = 85, 3.6% of all mosquito specimens) but most diverse host group (23 species and one unspecified taxa).

**Figure 4:**
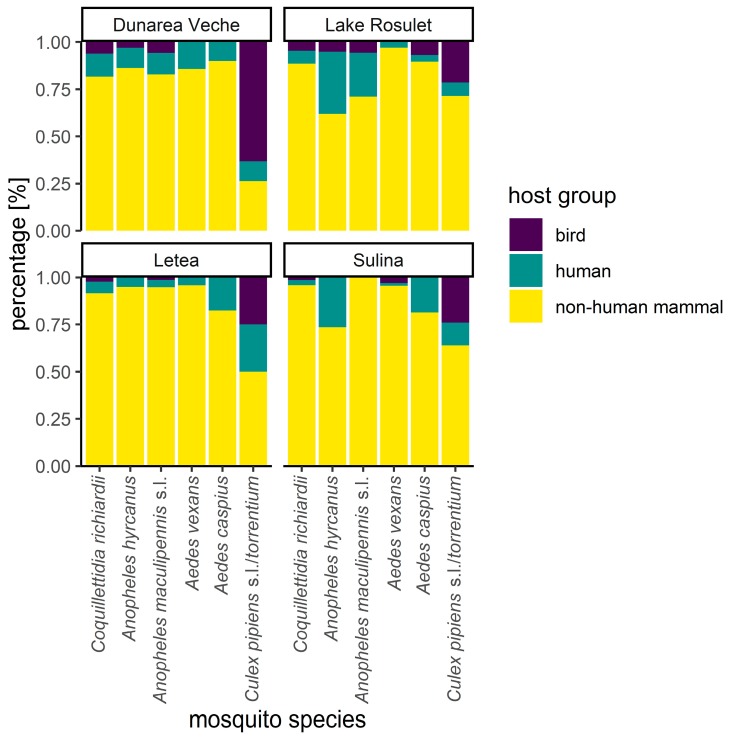
Percentage of host-feeding groups (birds, human, non-human mammals) of the six most abundant blood-fed mosquito species for two sampling sites in a rural/urban environment (Letea, Sulina) and two near-natural sampling sites (Dunărea Veche and Lake Roșuleț) in the DDBR, Romania (2014–2016).

These three most common host groups were determined for all six most abundant mosquito species (Figure 4). The ratios of the three host groups were statistically significantly (χ2 = 252.72, df = 10, p < 0.001) different between the six most abundant mosquito species. All five taxa except *Cx. pipiens* s.l./*torrentium* showed similar host-feeding patterns with clear preference for non-human mammals (81.2–95.7% of detected blood meal sources), followed by the host groups human and bird with 3.5–16.7% and 0.9–3.9%, respectively. However, *Ae. vexans* had statistically significantly different host group proportions with a higher proportion of non-human mammals compared to *An. hyrcanus*, *An. maculipennis* s.l. and *Ae. caspius* (adjusted p values < 0.05, Table S4). Similarly, we observed a higher proportion of non-human mammals for *An. maculipennis* s.l. and *An. hyrcanus* compared to *Cq. richiardii* (adjusted p values < 0.01, Table S4). Furthermore, the host-feeding pattern of *Cx. pipiens* s.l./*torrentium* was found to be statistically different (adjusted p values < 0.001, Supplementary Table S4) compared to all other five abundant species, with 53.2% non-human mammals, followed by 35.5% birds and 11.3% human.

The sampling sites had statistically significant different compositions of detected host groups (Figure 4, χ2 = 114.41, df = 6, p < 0.001). There were no statistical differences between the two near-natural sites Dunărea Veche and Lake Roșuleț (adjusted p values > 0.05, Table S5), but all other combinations were statically significantly different (adjusted p values < 0.05, Table S5, Figure S3). We observed higher percentages of non-human mammals and lower percentages of humans for the two more anthropogenic influenced sites, Letea and Sulina. Furthermore, we detected higher relative proportions of birds and humans for Sulina compared to Letea.

## 4. Discussion

In this study, we elucidated the possible origin, pattern of spatial-temporal dynamics, and eco-epidemiological factors of WNV in the ecosystem of DDBR. Our phylogeographic analysis identified at least two distinct introduction events of WNV lineage 2 to Romania. It circulates under a number of different virus variants (EEC1 and WEC1) with South Africa/Senegal and Greece as a possible hub for the progenitor of WNV strains involved in the outbreaks in Romania. The presence of a geographically distinct WNV clade (WEC1) is likely due to very recent introduction, adaptation to the local ecological conditions and some geographic barriers such as climate, vegetation, and vector species. Furthermore, the long-term circulation (EEC1) and adaptation of the virus to the host populations and its enzootic maintenance lead to spread into new geographic regions and local virus variants (in situ evolution).

Although the overlap between the phylogenetic and geographical clustering of the Romanian and Russian members of the Eastern European clade of WNV lineage 2 was expected, it is interesting to note that the clade also contains an Italian strain. This suggests a new, independent introduction of the EEC1 in the south-central part of the continent. Similarly to Eastern Europe, the Italian Peninsula is crossed by major Afro-European bird migration routes. To date, the dispersion pattern of WNV into temperate Eurasia can be best explained by bird migration [59,60,61,62], with short-distance migratory species as potential mode of WNV spread within Europe [62]. Interestingly, we found evidence of adaptive evolution in the WNV from Danube Delta also in non-structural genes, which likely indicates that the host immune selection pressure does not cause increases in viral fitness [63]. Mutations observed at amino acid positions T108I (C), S196P and R361K (E), I1192V (NS2A) and G2932R (NS5) have been found to be involved in the formation of EEC1. Although the impact of these mutations mostly from the nonstructural genes is unclear (likely occurred due to introduction of WNV in this country), similar changes modulated the host antiviral response by inhibition of interferon signaling [64]. The residue alternations R851K (NS1), I1462M (NS2B), R1516K (NS3), T2296A (NS4), N2305S (NS4) and R2719K (NS5) are specific for African variants. Similar patterns of convergent evolution have been described for WNV and suggest that a limited number of residue changes are permitted due to functional constraints [65].

This study successfully used a xenosurveillance approach to monitor the presence of WNV-specific antibodies in different host species. As demonstrated previously [66], mosquito-based surveillance allows non-invasive blood-sampling from free-roaming vertebrate hosts (e.g., feral horse) and from species which are rare or have a cryptic behavior (e.g., raccoon dog (*Nyctereutes procyonoides*), European mink (*Mustela lutreola*), Eurasian otter (*Lutra lutra*) or Golden jackal (*Canis aureus*)). This study supports previous studies, which identified horses and dogs as suitable sentinel species for WNV [53,54]. WNV seroprevalence in dogs (6.3%) and horses (3.1%) was similar to conventional sampling of the species in different areas with WNV activity [67,68,69]. Nevertheless, the seroprevalence in horses was markedly lower than in southeastern Romania (15.1%) [70]. WNV-specific antibodies were found in blood meals from horses and dogs in all four sampling sites, but not in mosquito blood-meals from human or bird. Thus, this indicate widespread, continuous WNV circulation, but probably only on a low level. In addition, due to potential cross-reactivity of the applied serological assay, we cannot exclude the possibility that one of the samples was also positive for USUV, a virus with a similar transmission cycle to WNV.

Feral horses and free-ranging cattle were the two most commonly detected host species, accounting for more than 50% of the detected hosts. These animals have their origins in pre-1990 state-owned collective farms and private homesteads from where they were released in more recent years. There is no official census published, but it is estimated that 4000 horses and a few other thousand cattle roam and reproduce freely in the DDBR. The high abundance in combination with the relatively huge body size [71] might explain why both host species are facilitated so often. However, there were differences between the sampling sites. Non-human mammals dominated the detected hosts for the two sampling sites located in the interface between anthropic and natural landscapes (Sulina and Letea), i.e., homesteads in direct proximity of livestock. In contrast, the other two near-natural sampling sites (Lake Roșuleț and Dunărea Veche) were both located deep inside the Danube Delta and only insignificantly anthropogenically influenced. However, humans are commonly present in a fishing cabin and an agricultural holding. In the absence of high abundances of cattle and horse, mosquitoes might rather select other available hosts, e.g., birds and humans. Thus, host availability probably is a decisive factor for the host-selection of mosquitoes, influencing the risk of local pathogen transmission.

At the same time, this study highlights the importance of *Cx. pipiens* s.l./*torrentium* as a WNV vector in Europe [72]. The only two WNV positive pools belonged to this taxon. Previous studies described the species complex as predominantly ornithophilic [30,31,32,60,73]. However, this study again demonstrates its catholic host-feeding pattern. As the other five most abundance taxa, *Cx. pipiens* s.l./*torrentium* predominantly fed on non-human mammals and humans but had the highest proportion of birds, i.e., a more than nine times higher proportion, making the species a potential bridge vector. This is in line with studies from Africa [74], Middle East [75], Europe [37], and North America [76]. Although several other collected mosquito species (e.g., *An. hyrcanus*) have been found positive for WNV-RNA in Romania [11,13,24], members of the *Culex pipiens* complex are considered the main vectors for WNV in both urban and rural/natural transmission cycles [13].

Active WNV circulation in the DDBR is strongly implicated by WNV infections of unfed mosquito specimens and serological evidence of WNV-specific antibodies in the hosts. However, this study collected only few specimens of the most competent vector *Cx. pipiens* s.l./*torrentium*. In previous studies in the DDBR [24,77], >95% of mosquitoes captured with bird-baited traps belonged to this species complex, while the most abundant species in our study (*Cq. richiardii*) was absent. The usage of a single type of trap in only a few sampling sites across the delta’s heterogenous landscape is likely to have contributed to a biased sampling outcome [78,79]. In addition, mobovirus transmission is generally restricted to small foci in non-epidemic years [80]. Therefore, the presence of WNV can be underestimated depending on the number, type and location of traps. Further studies are needed to identify and further understand the driving factors of landscape and time.

## 5. Conclusions

The detection of WNV-RNA and WNV-specific antibodies confirms the circulation of this important mobovirus in the DDBR. Serological evidence for WNV circulation confirms the applicability of mosquito-based surveillance in sero-epidemiological studies. Host identification for blood-fed mosquitoes allows the usage of host-specific conjugates. In addition, host-feeding patterns of *Cx. pipiens* s.l./*torrentium* underly the relevance of the taxon as an enzootic and bridge vector for WNV in Europe, which was further confirmed by the detection of WNV lineage 2 RNA in two pools of unfed specimens from the same taxon. Local overwintering or reintroduction of the virus could be considered decisive factors for the evolution, dispersal and endemisation of WNV in temperate Europe. Thus, to better understand the impact of ecological/immunological factors on WNV evolution, studies based on more comprehensive genetic data, including those from previously unsampled geographic regions, are required.

## Figures and Tables

**Table 1:** Samples of blood-fed mosquito species positive for West Nile virus-specific IgG and IgY antibodies with information on the host species, mosquito species, sampling site with the respective number of tested mosquito specimens (in brackets).

Host-Species	Mosquito Species	Dunărea Veche	Lake Roșuleț	Letea	Sulina	Sum
dog	*Aedes caspius*	1 (1)	0 (0)	0 (2)	0 (1)	1 (4)
	*Aedes vexans*	0 (1)	0 (3)	0 (1)	0 (2)	0 (7)
	*Anopheles hyrcanus*	0 (2)	1 (2)	1 (5)	1 (1) ^1^	3 (10)
	*Anopheles maculipennis* s.l.	0 (3)	1 (13)	0 (2)	0 (1)	1 (19)
	*Coquillettidia richiardii*	0 (5)	1 (40)	0 (7)	1 (10)	2 (62)
	*Culex modestus*	0 (0)	0 (1)	0 (0)	0 (0)	0 (1)
	*Culex pipiens* s.l./*torrentium*	0 (1)	0 (5)	0 (0)	0 (2)	0 (8)
horse	*Aedes caspius*	0 (2)	0 (6)	0 (10)	0 (7)	0 (25)
	*Aedes cinereus*	0 (0)	0 (0)	0 (2)	0 (0)	0 (2)
	*Aedes detritus*	0 (0)	0 (0)	0 (0)	0 (1)	0 (1)
	*Aedes vexans*	0 (0)	1 (20)	0 (92)	0 (8)	1 (120)
	*Anopheles algeriensis*	0 (0)	0 (0)	0 (1)	0 (1)	0 (2)
	*Anopheles hyrcanus*	0 (1)	3 (6)	0 (65)	0 (6)	3 (78)
	*Anopheles maculipennis* s.l.	0 (0)	0 (1)	1 (12)	1 (8)	2 (21)
	*Coquillettidia richiardii*	1 (2)	1 (32)	3 (90)	1 (16)	6 (140)
	*Culex pipiens* s.l./*torrentium*	0 (0)	0 (0)	0 (1)	0 (1)	0 (2)
human	*Aedes caspius*	0 (1)	0 (1)	0 (7)	0 (5)	0 (14)
	*Aedes flavescens*	0 (1)	0 (1)	0 (0)	0 (0)	0 (2)
	*Aedes vexans*	0 (1)	0 (1)	0 (5)	0 (1)	0 (8)
	*Anopheles algeriensis*	0 (0)	0 (1)	0 (0)	0 (8)	0 (9)
	*Anopheles hyrcanus*	0 (7)	0 (96)	0 (20)	0 (9)	0 (132)
	*Anopheles maculipennis* s.l.	0 (4)	0 (33)	0 (3)	0 (0)	0 (40)
	*Coquillettidia richiardii*	0 (12)	0 (28)	0 (11)	0 (4)	0 (55)
	*Culex modestus*	0 (0)	0 (1)	0 (0)	0 (2)	0 (3)
	*Culex pipiens* s.l./*torrentium*	0 (2)	0 (1)	0 (1)	0 (3)	0 (7)
	*Uranotaenia unguiculata*	0 (1)	0 (0)	0 (0)	0 (0)	0 (1)
bird	*Aedes caspius*	0 (0)	0 (2)	0 (0)	0 (0)	0 (2)
	*Aedes vexans*	0 (0)	0 (0)	0 (0)	0 (2)	0 (2)
	*Anopheles hyrcanus*	0 (2)	0 (15)	0 (0)	0 (0)	0 (17)
	*Anopheles maculipennis* s.l.	0 (2)	0 (8)	0 (1)	0 (0)	0 (11)
	*Coquillettidia richiardii*	0 (6)	0 (19)	0 (4)	0 (2)	0 (31)
	*Culex pipiens* s.l./*torrentium*	0 (12)	0 (3)	0 (1)	0 (6)	0 (22)
	Sum	2 (69)	8 (339)	5 (343)	4 (107)	19 (858)

^1^also positive for USUV-specific IgG.

**Table 2:** Frequency and percentage (in brackets) of detected host taxa for the six most abundant species and information on the overall proportion of each host.

	*Coquillettidia richiardii*	*Anopheles hyrcanus*	*Anopheles maculipennis* s.l.	*Aedes vexans*	*Aedes caspius*	*Culex pipiens* s.l./*torrentium*	Sum
*Anas platyrhynchos*		3 (0.4)	1 (0.4)		1 (0.9)		5 (0.2)
Anatidae	1 (0.1)	12 (1.5)	4 (1.4)		1 (0.9)		18 (0.8)
*Ardea purpurea*	10 (1.2)		1 (0.4)				11 (0.5)
*Circus aeroginosus*			1 (0.4)				1 (0)
*Corvus corone*	1 (0.1)						1 (0)
*Corvus fragilegus*				1 (0.4)			1 (0)
*Cyanistes caeruleus*	5 (0.6)					3 (5.4)	8 (0.3)
*Cygnus olor*	1 (0.1)						1 (0)
*Dendrocopos syriacus*	1 (0.1)						1 (0)
*Egretta garzetta*	1 (0.1)						1 (0)
*Falco tinnunculus*						1 (1.8)	1 (0)
*Gallus gallus*	1 (0.1)			1 (0.4)			2 (0.1)
*Hirundo rustica*	2 (0.2)						2 (0.1)
*Ixobrychus minutus*						3 (5.4)	3 (0.1)
*Locustella luscinoides*						3 (5.4)	3 (0.1)
*Motacilla alba*	1 (0.1)						1 (0)
*Netta rufina*	1 (0.1)						1 (0)
*Nycticorax nycticorax*	4 (0.5)	2 (0.3)	2 (0.7)			7 (12.5)	15 (0.6)
*Parus major*						2 (3.6)	2 (0.1)
*Pelecanus onocrotalus*	1 (0.1)		1 (0.4)				2 (0.1)
*Phalacrocorax carbo*	1 (0.1)						1 (0)
*Streptopelia orientalis*			1 (0.4)				1 (0)
*Strix aluco*						2 (3.6)	2 (0.1)
*Upupa epops*						1 (1.8)	1 (0)
*Homo sapiens*	55 (6.7)	132 (16.7)	40 (14.3)	8 (3.5)	14 (13.2)	7 (12.5)	271 (11.5)
*Bos taurus*	185 (22.4)	515 (65.1)	157 (56.1)	78 (33.9)	46 (43.4)	17 (30.4)	1009 (43)
Bovidae	2 (0.2)						2 (0.1)
*Canis aureus*	1 (0.1)	1 (0.1)			1 (0.9)		3 (0.1)
*Canis lupus*	62 (7.5)	10 (1.3)	19 (6.8)	7 (3)	4 (3.8)	8 (14.3)	111 (4.7)
*Capra hircus*	1 (0.1)	1 (0.1)					2 (0.1)
*Capreolus capreolus*	1 (0.1)		1 (0.4)				2 (0.1)
Chiroptera	2 (0.2)	1 (0.1)	1 (0.4)				4 (0.2)
*Equus caballus*	140 (16.9)	78 (9.9)	21 (7.5)	120 (52.2)	25 (23.6)	2 (3.6)	391 (16.7)
*Erinaceus europaeus*	1 (0.1)						1 (0)
*Felis catus*	34 (4.1)	3 (0.4)	7 (2.5)			1 (1.8)	47 (2)
*Lepus europaeus*	3 (0.4)			1 (0.4)	1 (0.9)	1 (1.8)	6 (0.3)
*Lutra lutra*	2 (0.2)	1 (0.1)					3 (0.1)
*Microtus levis*						1 (1.8)	1 (0)
*Mustela lutreola*	1 (0.1)						1 (0)
*Mustela nivalis*			1 (0.4)				1 (0)
*Nyctereutes procyonoides*	1 (0.1)						1 (0)
*Ovis aries*	8 (1)	4 (0.5)		2 (0.9)	2 (1.9)		16 (0.7)
*Pipistrellus kuhlii*	1 (0.1)						1 (0)
*Rattus norvegicus*	4 (0.5)					1 (1.8)	7 (0.3)
*Rhinolophus hipposideros*		1 (0.1)					1 (0)
*Sus scrofa*	299 (36.2)	28 (3.5)	25 (8.9)	12 (5.2)	11 (10.4)	2 (3.6)	382 (16.3)
blood-fed specimens	1054	1454	568	343	234	88	3741
succesful analyzed specimens ^1^	827	791	280	230	106	56	2290
identified hosts per mosquito species ^1^	834	792	283	230	106	62	2307
identified host taxa	30	13	15	9	9	17	

^1^differences between the number of successful analysed mosquito specimens and identified hosts results from a total of 17 mixed blood-meals.

## Supplementary Materials

The following are available online at https://www.mdpi.com/1999-4915/11/12/1159/s1, Figure S1: Schematic representation of the nucleotide (a) and amino acid (b) differences between WNV strains from Danube Delta generated during this study, Figure S2: Schematic representation of the specific amino acid replacements in the Danube Delta WNV genomes, Figure S3: Percentage of host-feeding groups (birds, human, non-human mammals) for the four sampling sites in the DDBR, Romania (2014–2016), Table S1: Frequency and percentage of mosquitoes collected at four sampling sites in the DDBR, Romania (2014–2016), Table S2: Samples of blood-fed mosquito species positive for West Nile virus-specific IgG antibodies with information on the host species, mosquito species, sampling year collected at four sampling sites in the DDBR, Romania (2014–2016), Table S3: Number and percentage (in brackets) of detected host taxa for the six less abundant species, Table S4: Results of Chi-square tests for the comparison of host-feeding groups between the six most abundant mosquito species with adjusted p-values collected at four sampling sites in the DDBR, Romania (2014–2016), Table S5: Results of Chi-square tests for the comparison of host-feeding groups of mosquitoes between the four sampling sites in the DDBR, Romania (2014–2016) with adjusted p-values.
