# Supplementary material for: Ecology of West Nile Virus in the Danube Delta, Romania: Phylogeography, Xenosurveillance and Mosquito Host-Feeding Patterns
Source: Viruses. 2019 Dec 14;11(12):1159. doi: 10.3390/v11121159 (PMC6950446; doi:10.3390/v11121159)
Supplement: Supplementary file 1 [file viruses-11-01159-s001.zip › Supplementary Figure 2.pdf]

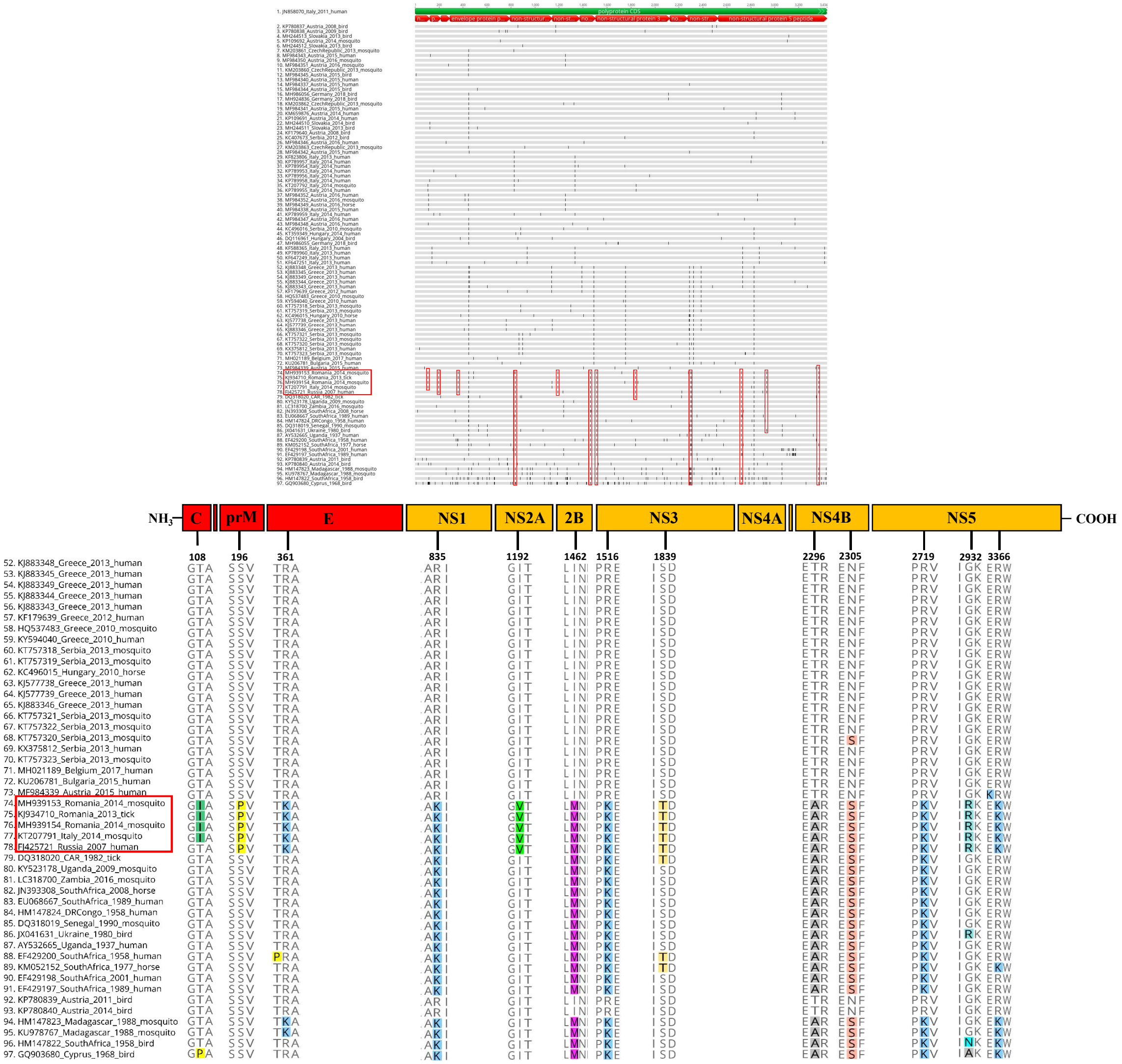

Figure S2. Schematic representation of the specific amino acid replacements in the Danube Delta WNV genomes. A number indicating the position of amino acid mutations in the gene and the single-letter amino acid codes are used to denote the EEC1 clade specific mutations and those involved in the convergent evolution along the polyprotein genes.
