# Supplementary material for: Ecology of West Nile Virus in the Danube Delta, Romania: Phylogeography, Xenosurveillance and Mosquito Host-Feeding Patterns
Source: Viruses. 2019 Dec 14;11(12):1159. doi: 10.3390/v11121159 (PMC6950446; doi:10.3390/v11121159)
Supplement: Supplementary file 1 [file viruses-11-01159-s001.zip › Supplementary Figure 3.pdf]

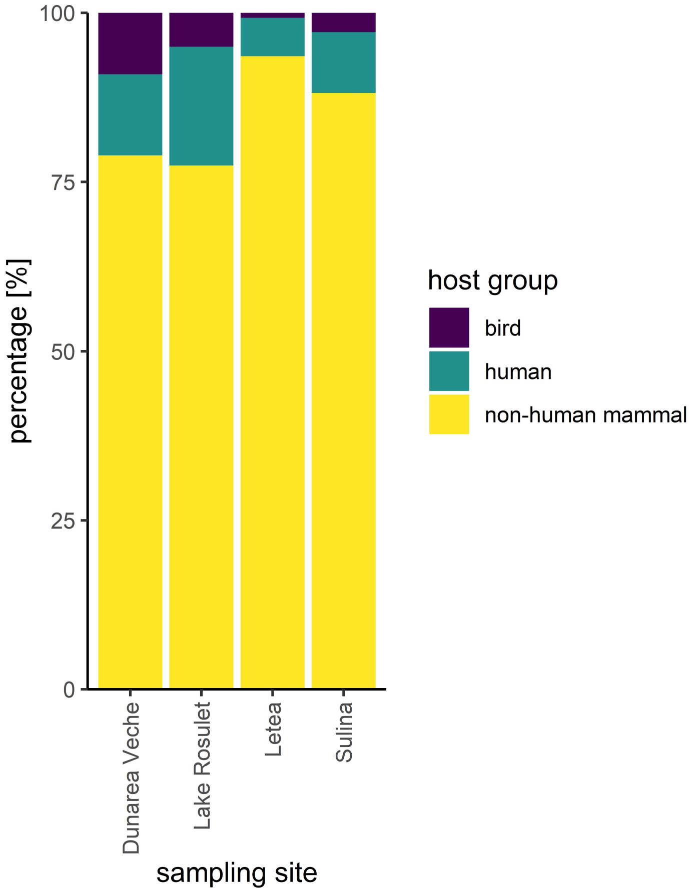

Figure S3. Percentage of host-feeding groups (birds, human, non-human mammals) for the four sampling sites in the DDBR, Romania (2014-2016). Two sampling sites in a rural/urban environment (Letea, Sulina) and two near-natural sampling sites (Dunărea Veche and Lake Roșuleț).
