# Supplementary material for: Ecology of West Nile Virus in the Danube Delta, Romania: Phylogeography, Xenosurveillance and Mosquito Host-Feeding Patterns
Source: Viruses. 2019 Dec 14;11(12):1159. doi: 10.3390/v11121159 (PMC6950446; doi:10.3390/v11121159)
Supplement: Supplementary file 1 [file viruses-11-01159-s001.zip › Supplementary Table S1.pdf]

Table S1. Frequency and percentage of mosquitoes collected at four sampling sites in the DDBR, Romania (2014-2016).

Mosquito species	Sum	Percentage
Coquillettidia richiardii	379,513	57.8820
Anopheles hyrcanus	162,725	24.8182
Anopheles maculipennis s.l.	28,363	4.3258
Aedes caspius	25,767	3.9299
Culex pipiens s.l./*torrentium*	23,134	3.5283
Aedes vexans	16,673	2.5429
Culex modestus	15,135	2.3083
Anopheles algeriensis	2,280	0.3477
unidentified	1,041	0.1588
Aedes flavescens	529	0.0807
Aedes detritus	246	0.0375
Aedes spec.	169	0.0258
Aedes cinereus	51	0.0078
Culex spec.	17	0.0026
Culiseta annulata	12	0.0018
Aedes hungaricus	4	0.0006
Anopheles spec.	3	0.0005
Uranotaenia unguiculata	3	0.0005
Aedes geniculatus	1	0.0002
Aedes sticticus	1	0.0002
