# Supplementary material for: Ecology of West Nile Virus in the Danube Delta, Romania: Phylogeography, Xenosurveillance and Mosquito Host-Feeding Patterns
Source: Viruses. 2019 Dec 14;11(12):1159. doi: 10.3390/v11121159 (PMC6950446; doi:10.3390/v11121159)
Supplement: Supplementary file 1 [file viruses-11-01159-s001.zip › Supplementary Table S2.pdf]

**Table S2.** Samples of blood-fed mosquito species positive for West Nile virus-specific IgG antibodies with information on the host species, mosquito species, sampling year collected at four sampling sites in the DDBR, Romania (2014-2016).

| **Host-species** | **Mosquito species** | **Year** | **Dunărea Veche** | **Lake Roșuleț** | **Letea** | **Sulina** | **Sum** |
|---|---|---|---|---|---|---|---|
| horse | *Aedes vexans* | 2016 | | 1 | | | 1 |
| | *Anopheles hyrcanus* | 2016 | | 3 | | | 3 |
| | *Anopheles maculipennis* s.l. | 2015 | | | 1 | 1 | 2 |
| | *Coquillettidia richiardii* | 2015 | | | 3 | 1 | 4 |
| | | 2016 | 1 | 1 | | | 2 |
| dog | *Aedes caspius* | 2016 | 1 | | | | 1 |
| | *Anopheles hyrcanus* | 2014 | | | 1 | | 1 |
| | | 2016 | | 1 | | 1[1] | 2 |
| | *Anopheles maculipennis* s.l. | 2014 | | 1 | | | 1 |
| | *Coquillettidia richiardii* | 2014 | | | | 1 | 1 |
| | | 2015 | | 1 | | | 1 |
| | Sum | | 2 | 8 | 5 | 4 | 19 |

[1]also positive for USUV-specific IgG
