# Supplementary material for: Ecology of West Nile Virus in the Danube Delta, Romania: Phylogeography, Xenosurveillance and Mosquito Host-Feeding Patterns
Source: Viruses. 2019 Dec 14;11(12):1159. doi: 10.3390/v11121159 (PMC6950446; doi:10.3390/v11121159)
Supplement: Supplementary file 1 [file viruses-11-01159-s001.zip › Supplementary Table S3.pdf]

Table S3. Frequency and percentage (in brackets) of detected host taxa for the six less abundant species.

	Anopheles algeriensis	*Culex modestus*	*Aedes flavescens*	*Aedes detritus*	*Aedes cinereus*	*Uranotaenia unguiculata*
Homo sapiens	9 (50)	3 (37.5)	2 (33.3)			1 (100)
Bos taurus	5 (27.8)	1 (12.5)		4 (80)	1 (33.3)	
Canis lupus		1 (12.5)				
Equus caballus	2 (11.1)			1 (20)	2 (66.7)	
Felis catus	2 (11.1)					
Rattus norvegicus		2 (25)				
Sus scrofa		1 (12.5)	4 (66.7)			
blood-fed specimens	29 (0.8)	36 (0.9)	7 (0.2)	6 (0.2)	3 (0.1)	1 (<0.1)
succesful analysed specimens	18	8	6	5	3	1
identified hosts per mosquito species	18	8	6	5	3	1
identified host taxa	4	5	2	2	2	1
