# Supplementary material for: Ecology of West Nile Virus in the Danube Delta, Romania: Phylogeography, Xenosurveillance and Mosquito Host-Feeding Patterns
Source: Viruses. 2019 Dec 14;11(12):1159. doi: 10.3390/v11121159 (PMC6950446; doi:10.3390/v11121159)
Supplement: Supplementary file 1 [file viruses-11-01159-s001.zip › Supplementary Table S4.pdf]

Table S4. Results of Chi-square tests for the comparison of host-feeding groups between the six most abundant mosquito species with adjusted p-values collected at four sampling sites in the DDBR, Romania (2014-2016).

Mosquito species	Mosquito species	adjusted p value	χ^2
Aedes caspius	*Aedes vexans*	0.036648349	12.0288742
Aedes caspius	*Anopheles hyrcanus*	1	0.876218151
Aedes caspius	*Anopheles maculipennis* s.l.	1	1.050654796
Aedes caspius	*Coquillettidia richiardii*	0.5154945	6.741357691
Aedes caspius	*Culex pipiens* s.l./*torrentium*	1.88E-07	36.3867405
Aedes vexans	*Anopheles hyrcanus*	9.19E-06	28.61119323
Aedes vexans	*Anopheles maculipennis* s.l.	0.00018112	22.64880073
Aedes vexans	*Coquillettidia richiardii*	0.227764729	8.374984545
Aedes vexans	*Culex pipiens* s.l./*torrentium*	1.80E-18	87.13728235
Anopheles hyrcanus	*Anopheles maculipennis* s.l.	1	3.276589246
Anopheles hyrcanus	*Coquillettidia richiardii*	8.18E-09	42.65875941
Anopheles hyrcanus	*Culex pipiens* s.l./*torrentium*	2.16E-31	146.6318183
Anopheles maculipennis s.l.	*Coquillettidia richiardii*	0.006237562	15.57043205
Anopheles maculipennis s.l.	*Culex pipiens* s.l./*torrentium*	2.48E-12	58.85872968
Coquillettidia richiardii	*Culex pipiens* s.l./*torrentium*	3.07E-23	109.094442
