# Supplementary material for: Ecology of West Nile Virus in the Danube Delta, Romania: Phylogeography, Xenosurveillance and Mosquito Host-Feeding Patterns
Source: Viruses. 2019 Dec 14;11(12):1159. doi: 10.3390/v11121159 (PMC6950446; doi:10.3390/v11121159)
Supplement: Supplementary file 1 [file viruses-11-01159-s001.zip › Supplementary Table S5.pdf]

Table S5. Results of Chi-square tests for the comparison of host-feeding groups of mosquitoes between the four sampling sites in the DDBR, Romania (2014-2016) with adjusted p-values.

Sampling site	Sampling site	adjusted p value	χ^2
Dunărea Veche	Lake Roșuleț	0.06759567	8.97194151
Dunărea Veche	Letea	6.31E-14	64.3731036
Dunărea Veche	Sulina	0.00866489	13.0804717
Lake Roșuleț	Letea	1.09E-19	90.9134496
Lake Roșuleț	Sulina	0.00058796	18.4612242
Letea	Sulina	0.00932428	12.9337851
