# Supplementary figures and images for: Ecology of West Nile Virus in the Danube Delta, Romania: Phylogeography, Xenosurveillance and Mosquito Host-Feeding Patterns
Source: Viruses. 2019 Dec 14;11(12):1159. doi: 10.3390/v11121159 (PMC6950446; doi:10.3390/v11121159)

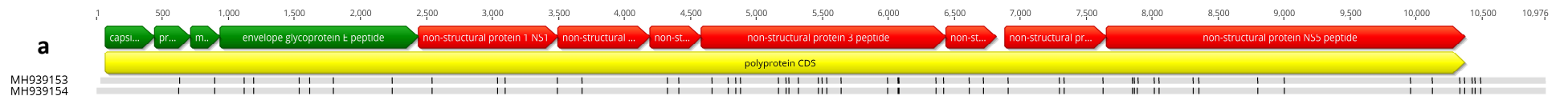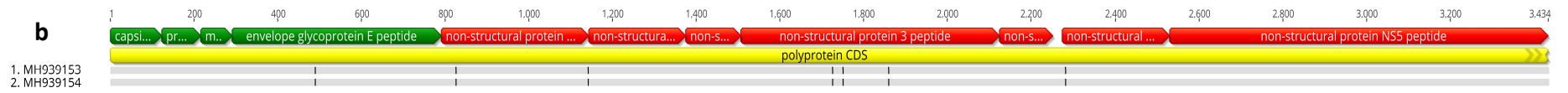

Supplement: Supplementary file 1 [file viruses-11-01159-s001.zip › Supplementary Figure 1.pdf]
